# The effect of dancesport in psychological well-being of college students: chain mediation of perceived social support and study engagement

**DOI:** 10.3389/fpsyg.2025.1699654

**Published:** 2025-12-08

**Authors:** Zitong Chen, Shuo Wang, Xianglong Jiang, Tongtong Che, Yan Shi

**Affiliations:** 1 Jiangsu Vocational College of Agriculture and Forestry, Jurong, China; 2 Hebei Sport University, Shijiazhuang, China; 3 Qingdao University, Qingdao, China; 4 Capital University of Physical Education and Sports, Beijing, China

**Keywords:** dancesport, psychological well-being, perceived social support, study engagement, mediation effects

## Abstract

**Objective:**

Promoting the psychological well-being of college students is a critical issue in mental health education. While physical activity is known to enhance well-being, the specific mechanisms through which dancesport—an activity integrating sport, art, and social interaction—exerts its influence remain underexplored. This study investigates the chain mediation effect of perceived social support and study engagement in the relationship between dancesport participation and psychological well-being among college students.

**Methods:**

A cross-sectional study was conducted with 1,012 Chinese college students enrolled in dancesport courses. Participants completed self-report measures assessing dancesport participation, psychological well-being, perceived social support, and study engagement. Data were analyzed using structural equation modeling (SEM) and the SPSS PROCESS macro (Model 6) with 5,000 bootstrap samples to test the proposed chain mediation model.

**Results:**

The results indicated that: (1) Dancesport participation was significantly and positively associated with psychological well-being of college students: (*β* = 0.32, *p* < 0.001). (2) Perceived social support and study engagement play partial mediating roles between dancesport and psychological well-being, with indirect effects of 0.11 (95% CI [0.06, 0.17]) and 0.08 (95% CI [0.03, 0.13]), respectively. (3) A significant chain mediation pathway was identified: dancesport → perceived social support → study engagement → psychological well-being (indirect effect = 0.037, 95% CI [0.015, 0.062]).

**Conclusion:**

This study reveals that dancesport enhances college students’ psychological well-being not only directly but also indirectly by first fostering perceived social support, which in turn promotes greater study engagement. These findings underscore the multifaceted benefits of dancesport and provide a theoretical and practical basis for incorporating artistic and social physical activities into university mental health promotion programs.

## 1 Introduction

As the backbone of the youth group, the mental health of college students has a profound impact on social development. However, at present, college students generally face multiple challenges such as academic pressure, employment competition, interpersonal relationship conflicts and family conflicts, which are prone to triggering negative emotions such as anxiety and depression, with low levels of psychological well-being, and mental health problems are becoming increasingly prominent (Song et al., 2021). World Health Organization (2022) report shows that in 2019, about 970 million people worldwide suffer from mental disorders, and the incidence of anxiety and depression continues to climb. Focusing on the Chinese university student population, a survey by the Chinese Academy of Sciences indicates that career planning (e.g., further education and employment) is a core stressor, while lifestyle behaviors such as sleep quality and late-night habits also significantly affect mental health (Fang et al., 2008). Psychological well-being, as a core concept of positive psychology, emphasizes an individual’s comprehensive cognitive and emotional experience of the state of life, covering the dimensions of self-acceptance, life goals, and social connections, which is not only an objective reflection of psychological health, but also promotes the development of potential, and environmental adaptation and social integration (Yu, 2022; Seligman, 2011). Therefore, enhancing college students’ psychological well-being has become an important issue in mental health education. The present study focuses on the intervention role of dancesport and explores its influence mechanism on college students’ psychological well-being, aiming to provide theoretical and practical basis for the promotion of college students’ psychological health.

Research has shown that physical activity is a key factor in enhancing psychological well-being (Li and Liu, 2014). College students release stress and regulate emotions through exercise, which in turn enhances the experience of well-being (Zhao, 2020). The multidimensional characteristics of dancesport, as a form of exercise that integrates music, socialization, and aesthetics, may have a unique impact on psychological well-being: on the one hand, dancesport movements alleviate anxiety through the release of endorphins (Biddle and Asare, 2011), and in contrast to general exercise, the rhythmic and coordinative requirements specific to dancesport more significantly enhance prefrontal lobe activity in the brain (Kattenstroth, 2010), enhancing emotional regulation. The acquisition and performance of dancesport movements requires overcoming technical challenges, which is in line with the theory of “mastery experience” and strengthens self-efficacy through the accumulation of successful experiences (Bandura, 1997). On the other hand, the “pairs/groups” nature of dancesport (e.g., Latin and group dances) promotes social bonding (Cohen and Wills, 1985), and perceptions of social support are a core predictor of well-being (Thoits, 1983). Dancesport releases emotions through non-verbal art forms (Freud’s Sublimation Theory), and its aesthetic experience activates the brain’s reward circuitry, generating feelings of pleasure and meaning (Wang, 2014). In addition, the multidimensional integration of dancesport (physical + artistic + social) may have a superimposed effect on well-being compared to ordinary sports (e.g., running, fitness): For instance, Murcia et al. (2010) found that dancesport participants’ well-being was significantly higher than that of fitness participants, attributing this to its simultaneous satisfaction of needs for belonging (social) and creativity (artistic). Although extensive research has confirmed the general benefits of physical exercise for mental health, this study focuses specifically on “ballroom dancing.” As a comprehensive activity integrating sports, art, and social interaction, multiple studies indicate that ballroom dancing can more directly activate the brain’s reward system through multisensory stimulation, producing a unique “synergistic effect” on psychological well-being (Pinniger et al., 2012; Liu et al., 2021). Compared to conventional exercises like running and fitness training, the artistic nature and rhythmicity of dance sport can more effectively stimulate positive emotions, offering unique advantages in reducing anxiety, enhancing emotional regulation, and improving social skills (Koch et al., 2019). Its artistic expression—such as emotional elevation and aesthetic experiences through body language—and its inherent social interaction—like partner coordination in duets or group collaboration in ensemble dances—provide psychological nourishment pathways unavailable in ordinary exercise. It simultaneously fulfills individuals’ needs for belonging (social), aesthetic appreciation (art), and competence (skill mastery), aligning closely with the core psychological needs of self-determination theory. Therefore, exploring the mechanisms of sports dance not only deepens our understanding of the “integration of physical education and arts” model for mental health promotion but also offers a unique perspective for designing more effective comprehensive psychological intervention programs in higher education institutions. Based on this, this study proposes hypothesis H1: Dancesport participation is positively associated with college students’ psychological well-being.

Perceived social support refers to an individual’s perception and evaluation of the availability of external support (Ye, 2005; Haber et al., 2007), which is reflected in a positive psychological state. Participation in social activities (e.g., dancesport) enhances perceptions of social support through group interactions (Cohen and Wills, 1985), and high perceptions of social support can buffer stress, enhance emotional regulation (Thoits, 1983), and indirectly enhance well-being by satisfying psychological needs (Münster Halvari et al., 2010). Social support in dancesport is multidimensional: firstly, dancesport programs have stable social networks (e.g., partners, teachers), and secondly, body language between partners (e.g., lifting, coordination) builds trust (Wang, 2016) and provides emotional support. In addition, teacher feedback helps individuals to improve their movements and enhances the sense of “being recognized” (Zimmermann et al., 2024). According to a survey of Asian university students, for every 1-unit increase in perceived social support among group exercise participants, well-being increased by 0.32 standard deviations (*β* = 0.32, *p* < 0.01) (Kim et al., 2021). The indirect effect of social support between exercise participation and well-being was verified by structural equation modeling to account for 38% (Zhang and Kong, 2016). For example, support from peers or teachers can strengthen an individual’s sense of self-worth and motivation level (Smith, 1999), which in turn promotes well-being. Based on this, this study proposes hypothesis H2: Appreciating social support mediates the relationship between dancesport and psychological well-being.

Study engagement is a process of continuous emotional investment, covering the dimensions of vigor, dedication, and concentration (Schaufeli et al., 2002), and several studies have shown that study engagement is a positive predictor of well-being (Schaufeli et al., 2002; Yao, 2011). People with high study engagement are more likely to derive a sense of achievement from learning (e.g., acquiring new skills), which is consistent with the “achievement” dimension of the PERMA model. It encompasses five core elements: Positive Emotion, Engagement, Relationships, Meaning, and Accomplishment (Seligman, 2011). Study engagement is highly correlated with state of mindstream (concentration, distorted sense of time) (Schaufeli et al., 2002), which is a central source of well-being (Buchanan and Csikszentmihalyi, 1991). The mean correlation coefficient between study engagement and well-being was found to be r = 0.41 (95% CI [0.35, 0.47]) by integrating 12 studies (Gao, 2013). After 8 weeks of exercise intervention showed that the experimental group’s study engagement level increased by 23% and the increase in well-being was significantly higher than that of the control group (*p* < 0.05) (Yao, 2011). The artistic and fun nature of dancesport is in line with the principle of “interest stimulation” in self-determination theory (Deci and Ryan, 2000), which promotes active engagement (Fredrickson, 2001), and the ability to concentrate (e.g., memorizing movements and listening to the music) developed in dancesport training can be transferred to the academic scene (Schunk and Mullen, 2012), resulting in “positive feedback on mental toughness.” Based on this, this study proposes hypothesis H3: Study engagement is associated with the relationship between dancesport and psychological well-being in a mediating role.

**Figure 1:**
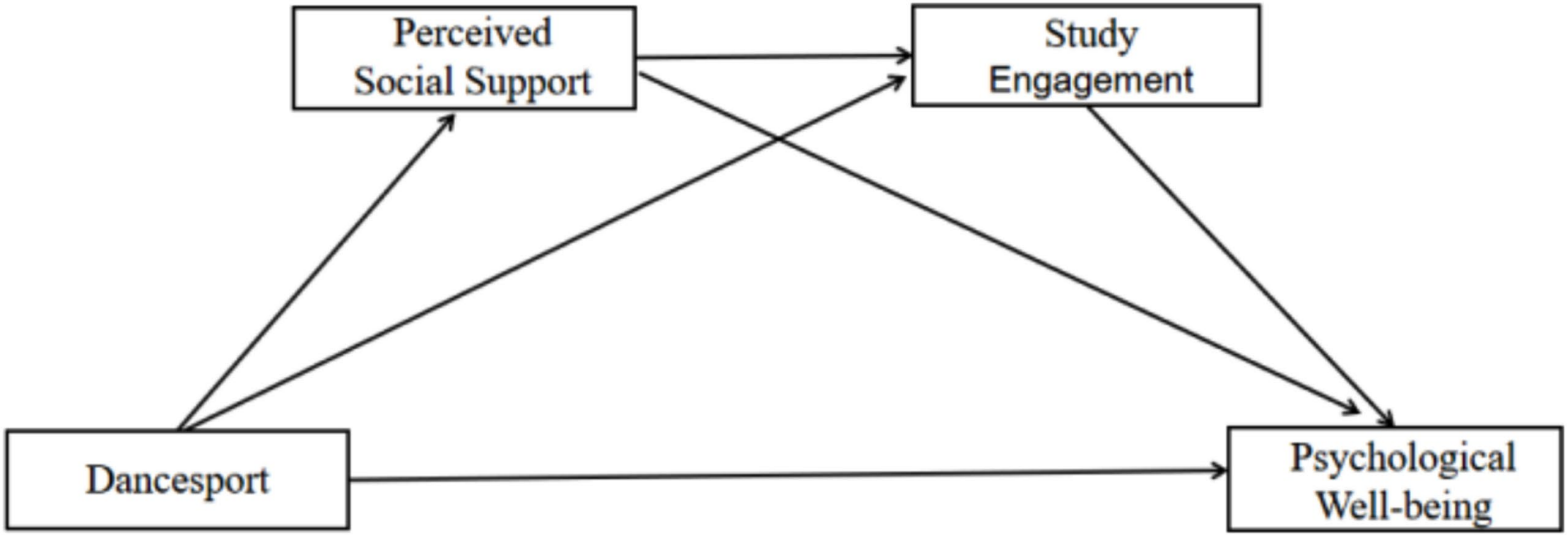
Chain mediation effect model for perceived social support and study engagement.

There may be a synergistic effect between social support and study engagement. According to resource preservation theory (Hobfoll, 2001), social support as a psychological resource can be transformed into psychological capital (e.g., confidence, perseverance) needed for learning (Hou, 2020), and social support perception is an antecedent variable of study engagement (Zhang and Wang, 2018). Individuals with high social support perceptions are more likely to develop positive coping strategies and stimulate intrinsic motivation to learn (Deci and Ryan, 2000), which enhances the level of study engagement, while social support indirectly facilitates study engagement by decreasing stress perceptions (e.g., academic anxiety). The “stress buffer model” states that high social supporters are more effective in regulating negative emotions and maintaining a focused state (Thoits, 1983). The multidimensional integrative nature of dancesport (art + social + exercise) may amplify the mediating effect compared to common sports: activating dopamine release through aesthetic experience, directly enhancing well-being (Wang, 2014). High-frequency interaction accelerates the accumulation of social support resources, forming a virtuous cycle of “comprehension of social support → study engagement → psychological well-being.” Based on this, this study proposes hypothesis H4: there is a chain mediation effect between appreciation of social support and study engagement in dancesport and psychological well-being. The hypothetical modeling study is shown in Figure 1.

## 2 Participants and methods

### 2.1 Participants

Convenience sampling method was used in this study. College students participating in dancesport courses in three colleges and universities in eastern China were sampled for the survey. It should be noted that this sampling strategy, while practical, focuses on students who have already elected to participate in dancesport, who may differ from the general student population in characteristics such as pre-existing interest in physical activity or baseline levels of social engagement. Subject respondents were informed and agreed to complete the questionnaire between June 25th and July 15th. Paper and online versions of the questionnaire were distributed to each individual, and the subjects completed the questionnaire under the guidance of the instructor. The experimenters collected and submitted 1,058 questionnaires on site. Afterwards, invalid questionnaires such as routine responses, incomplete demographic questionnaires were excluded. After analyzing the information and missing options in the questionnaires, the valid questionnaires were 1,012 with an effective rate of 95.7%. All participants are enrolled in university-offered ballroom dance courses, primarily focusing on the Cha Cha Cha partner dance within Latin dance, supplemented by group formation dance training. During instruction, students are assigned fixed practice partners and regularly engage in group presentations and classroom interactions. The curriculum mandates at least one organized partner practice session per week, ensuring both the frequency and depth of social interaction. The demographic information of the subjects is shown in Table 1. The studies involving humans were approved by the Ethics Committee of the St. Paul University Philippines (Approval No.: SPUP202306375RCZ). This research was conducted as part of the first author’s doctoral program at SPUP. The study procedures and ethical approval were also reviewed and endorsed for implementation by the administrative departments of the participating Chinese universities (Jiangsu Vocational College of Agriculture and Forestry, Qingdao University, and Hebei Sport University). The study was conducted in accordance with the local legislation and institutional requirements, as well as the tenets of the Declaration of Helsinki. This research was initiated as a doctoral project of the first author through St. Paul University Philippines. The ethical oversight was therefore primarily provided by the SPUP Ethics Committee, in line with standard academic protocols. The collaboration with and data collection at all Chinese university sites were conducted with the formal permission of the respective institutional authorities.

**Table 1:** Subjects’ demographic information Table (*n* = 1,012).

Characteristics	Category	Sample Size	Percentage
Gender	Male	387	38.24%
	Female	625	61.76%
Grade	Freshman	159	15.71%
	Sophomore	462	45.65%
	Junior	234	23.12%
	Senior	157	15.51%
Place of Origin	Urban Areas	554	54.74%
	Rural Areas	458	45.26%
One-child Family	Yes	569	56.23%
	No	443	43.77%

### 2.2 Measures

#### 2.2.1 Dancesport participation

Based on the multidimensional measurement theory of sport involvement (Godin and Shephard, 1985), this study self-administered the Dancesport Participation Scale, taking into account the special characteristics of dance activities (e.g., artistic, social). Referring to existing sport behavior scales (e.g., International Physical Activity Questionnaire IPAQ) and dance research tools (Quiroga Murcia and Kreutz, 2012), the question items were constructed from four dimensions: frequency of behavior, duration, intensity, and emotional involvement. The scale consists of 6 items and is scored on a 5-point Likert scale (1 = “not at all” to 5 = “completely”), with higher scores indicating greater participation. The specific dimensions and examples are as follows: frequency of behavior: “The number of times I participate in dancesport classes or self-directed practice per week is:” (1 = “less than 1 time,” 2 = “1 time,” 3 = “2 times“, 4 = “3 times“, 5 = “4 or more times”); Length of a single session: “The average length of time I participate in dancesport each time is:” (1 = “less than 30 min,” 2 = “30–60 min,” 3 = “60–90 min”, 4 = “90–120 min”, 5 = “more than 120 min”); Continuous Cycle: “The length of my continuous participation in dancesport is”: (1 = “less than 3 months,” 2 = “3–6 months,” 3 = “6–12 months”, 4 = “1–2 years”, 5 = “more than 2 years”); Intensity Perception: “During dance practice, I feel a greater physical load (e.g., sweating, faster breathing)”; Emotional involvement: “I give my full attention during dance and forget about the passage of time”; Social interaction: “I often practice with a partner or team member.” Three sport psychologists and two sport dancesport teachers were invited to review the items, and the revised content validity index (CVI) was 0.92. Structural validity: exploratory factor analysis (EFA) was conducted to extract two common factors (“behavioral involvement” and “psychological involvement”), with a cumulative variance explained of 68.5%, KMO = 0.86, and a significant Bartlett’s test (χ^2^ = 432.7, *df* = 15, *p* < 0.001). Reliability: the Cronbach’s *α* coefficient was 0.89 and the split-half reliability was 0.84.

#### 2.2.2 Psychological well-being

The Psychological Well-Being Scales (PWBS), developed by Ryff (1989) based on the “Multidimensional Theory of Psychological Well-Being,” is a well-established and widely used international scale. Its theoretical foundation integrates humanistic psychology and positive psychology, emphasizing that well-being is not only about emotional experiences, but also about individuals’ assessment of their self-realization of their potential and achievement of their life goals in six dimensions: autonomy, environmental mastery, personal growth, positive relations with others, purpose in life, and self-acceptance. Ryff and Keyes (1995) initially proposed a version of 84 questions, which was later simplified into shorter versions of 54 questions and 18 questions. The version chosen for this study was the 18-item short version (3 questions for each dimension), which was culturally adapted to the Chinese university student population (e.g., “pursuing personal goals” was changed to “pursuing academic and personal development goals”). Semantic consistency was ensured through back-translation. A 6-point Likert scale was used (1 = “not at all consistent” to 6 = “completely consistent”), with higher total scores indicating higher levels of psychological well-being. In this study, the Cronbach’s *α* coefficient for the scale was measured to be 0.85, indicating good internal consistency. The six-factor structure was validated by a confirmatory factor analysis (CFA), and the fitting of model indicators were met (χ^2^/df = 2.41, RMSEA = 0.07, CFI = 0.93).

#### 2.2.3 Perceived social support

This study used the Multidimensional Scale of Perceived Social Support (MSPSS), which was developed by Zimet et al. (1988), and is a classic instrument for measuring individuals’ subjective perceived social support levels. The scale is grounded in the social support buffer hypothesis (Cohen and Wills, 1985), which emphasizes the protective effect of an individual’s perception of support from family, friends, and significant others on mental health, with a total of 12 questions (4 questions per dimension). For the Chinese college student population, Jiang (1999) validated the reliability of the Chinese version of the MSPSS and adapted the scale to use a Likert 5-point scale (1 = “not at all consistent” to 5 = “completely consistent”) to adapt to local Higher scores indicate higher levels of perceived social support, and the Cronbach’s *α* coefficient for this scale in this study was 0.91. Confirmatory factor analysis (CFA) showed a good fit to the three-factor structure (χ^2^/df = 2.15, RMSEA = 0.06, CFI = 0.96).

#### 2.2.4 Study engagement

This study utilized the Utrecht Work Engagement Scale-Student (UWES-S), which was developed by Schaufeli et al. (2002) based on the Work Engagement Theory and was applied to the student population. The scale emphasizes three dimensions of study engagement: Vigour: a sense of energy and psychological resilience in learning: The three dimensions are Vigour (a sense of energy and psychological resilience in learning), Dedication (a sense of recognition of meaning and enthusiasm for learning), and Absorption (the state of being fully focused on the study task). For the Chinese context, Fang et al. (2008) validated a localized version, which involved simplifying the scale and adjusting the original 7-point Likert scale to a 5-point format (1 = “not at all” to 5 = “completely”) and simplifying the 17 questions to 14 questions to improve the applicability (Fang et al., 2008), with higher scores indicating higher levels of study engagement. The Cronbach’s α coefficient for the scale in this study was 0.88; Confirmatory factor analysis (CFA) supported the three-dimensional structure (χ^2^/df = 2.30, RMSEA = 0.05, CFI = 0.94).

### 2.3 Data analysis

This study utilized a quantitative analytical paradigm designed to test the chain-mediated mechanisms between dancesport participation, navigational social support, academic engagement, and psychological well-being. Data were analyzed using SPSS 26.0 and AMOS 24.0 software. Data characteristics were initially explored through common method bias tests, descriptive statistics, and Pearson correlation analysis between variables. During data collection, this study implemented multiple procedural controls to minimize common method bias: these included using anonymous questionnaires, incorporating reverse-scored items in certain scales, and strategically dispersing items measuring different constructs throughout the questionnaire to create psychological isolation. To verify the robustness of the chain mediated effect, Bootstrap sampling was performed 5,000 times using the PROCESS macro program (Model 6) developed by Hayes (2013) to calculate the confidence intervals for the indirect effect. Meanwhile, structural equation modeling (SEM) was constructed using AMOS, the construct validity of the measurement model was tested by validated factor analysis (CFA), and the goodness of fit of the structural model was assessed using maximum likelihood estimation. Model fit was judged by a combination of the following metrics: chi-square degrees of freedom ratio (χ^2^/df) less than 3, comparative fit index (CFI) greater than 0.90, and root mean square of the error of approximation (RMSEA) less than 0.08. All analyses controlled for demographic variables such as gender, grade level, place of origin, and one-child status to ensure the accuracy of the results. This comprehensive analytic strategy ensured the rigor of the model test and provided multi-method validation support for the chain mediation effect.

## 3 Research results

### 3.1 Common method bias testing

In the actual measurement program, all questionnaires were completed anonymously. Harman single-factor testing was used to test for common method bias, with 1 factor forced to be extracted, and the results showed that the explained variance of the first factor was 25.314%, which was lower than the critical criterion of 40% (Podsakoff et al., 2003), indicating that the common method bias was not serious. Data suitability was further verified by KMO test and Bartlett test of sphericity: the KMO value was 0.895 (>0.8) and the Bartlett test of sphericity value was 1350.159, *df* = 1,225 (corresponding to 50 question items), *p* < 0.001, indicating suitability for factor analysis. Subsequently, exploratory factor analysis (EFA) was performed on all variables, using principal component analysis with maximum variance rotation to extract eight factors with eigenroots greater than 1, with a cumulative variance explained of 67.8%. The first of these factors had an explained rate of 25.314%, with no single factor dominance. However, it is important to acknowledge that while Harman’s test is a widely used diagnostic technique, it has limitations and may not be sufficiently sensitive to detect all forms of common method variance. Furthermore, the use of self-report measures for all constructs remains susceptible to potential biases such as social desirability, which could inflate the relationships between variables.

### 3.2 Descriptive statistics and correlation analysis

Correlation analysis showed (Table 2) that gender, place of origin, and one-child family had a weak but significant effect on psychological well-being, so they were included as control variables in the model’s subsequent mediation and chain mediation effect analyses. Meanwhile there was a significant positive correlation between the four study variables of dancesport, perceived social support, study engagement and psychological well-being.

**Table 2:** Descriptive statistics and correlation analysis of variables.

Variables	M ± SD	Skewnes	Kurtosis	1	2	3	4	5	6	7
1. Dancesport	3.82 ± 0.71	−0.32	0.15							
2. Perceived social support	4.15 ± 0.63	−0.45	0.58	0.38**						
3. Study engagement	3.96 ± 0.58	−0.21	−0.06	0.35**	0.51**					
4. Psychological well-being	4.02 ± 0.66	−0.38	0.22	0.42**	0.47**	0.33**				
5. Gender				0.05	0.18**	0.08	0.15*			
6. Grade				0.04	0.07	0.12*	0.09	−0.03		
7. Place of Origin				0.03	0.06	−0.04	0.10*	0.05	0.08	
8. One-child family				0.02	0.11*	0.05	0.09*	0.12*	0.04	0.21

M, Mean; SD, Standard Deviation. Skewness and Kurtosis values indicate that the data for all variables approximate a normal distribution (absolute values < 2). *P < 0.05, **P < 0.01, ***P < 0.001; same below.

### 3.3 Mediation and chain mediation effect analysis

A combination of hierarchical multiple regression, stepwise test of mediating effects, Bootstrap sampling and chained path analysis were used to systematically validate the mechanism by which dancesport is associated with psychological well-being through social support and study engagement. The full structural equation model (SEM) explained a substantial proportion of the variance in psychological well-being (*R*^2^ = 0.36). The goodness-of-fit indices for the structural model indicated an acceptable to good fit to the data: χ^2^/df = 2.58, RMSEA = 0.059, CFI = 0.92, TLI = 0.90. The choice of methodology balances traditional statistical logic (e.g., Baron and Kenny steps) with modern robustness requirements (e.g., Bootstrap) to ensure the reliability and theoretical explanatory power of the results. The results showed (Table 3) that the direct association of dancesport with psychological well-being was significant (*β* = 0.32, *p* < 0.001), and that among the control variables, gender (female) and town origin significantly and positively predicted psychological well-being. Dancesport was significantly associated with higher perceived social support (*β* = 0.38, *p* < 0.001) and perceived social support was further significantly associated with psychological well-being (*β* = 0.29, *p* < 0.001). The dancesport component was indirectly associated with psychological well-being (*β* = 0.19, *p* = 0.007) through its association with study engagement (*β* = 0.12, *p* = 0.017). Perceived social support was significantly associated with study engagement (*β* = 0.51, *p* < 0.001), forming a chain path of “dancesport → perceived social support → study engagement → psychological well-being.”

**Table 3:** Regression analysis of the chain mediation model.

Dependent variable	Predictor Variable	*β*	SE	*t*	*p*	*R* ^2^	*F*
Psychological well-being							
Model 1(direct effect)	Dancesport	0.32	0.06	5.33	<0.001***	0.18	*F* = 12.73***
	Gender(Female = 1)	0.11	0.05	2.20	0.028*		
	Place of Origin(Urban Areas = 1)	0.09	0.04	2.25	0.025*		
Model 2 (+perceived social support)	Dancesport	0.24	0.06	4.00	<0.001***	0.27	*F* = 18.05***
	Perceived social support	0.29	0.07	4.14	<0.001***		
Model 3 (+study engagement)	Dancesport	0.18	0.06	3.00	0.003**	0.31	*F* = 20.89***
	Perceived Social Support	0.20	0.07	2.86	0.005**		
	Study Engagement	0.19	0.07	2.71	0.007**		
Perceived social support							
Model 4	Dancesport	0.38	0.05	7.60	<0.001***	0.21	*F* = 15.62***
Study engagement							
Model 5	Dancesport	0.12	0.05	2.40	0.017*	0.34	*F* = 25.10***
	Perceived social support	0.51	0.05	10.20	<0.001***		

*β* = standardized regression coefficient; SE = standard error; *t* = *t*-value; *p* = significance level. Control variables: Gender (1 = Female, 0 = Male); Place of Origin (1 = Urban, 0 = Rural). The overall structural equation model demonstrated good fit (χ^2^/df = 2.58, RMSEA = 0.059, CFI = 0.92, TLI = 0.90) and explained 36% of the variance in psychological well-being (*R*^2^ = 0.36), as reported in the text.

**Figure 2:**
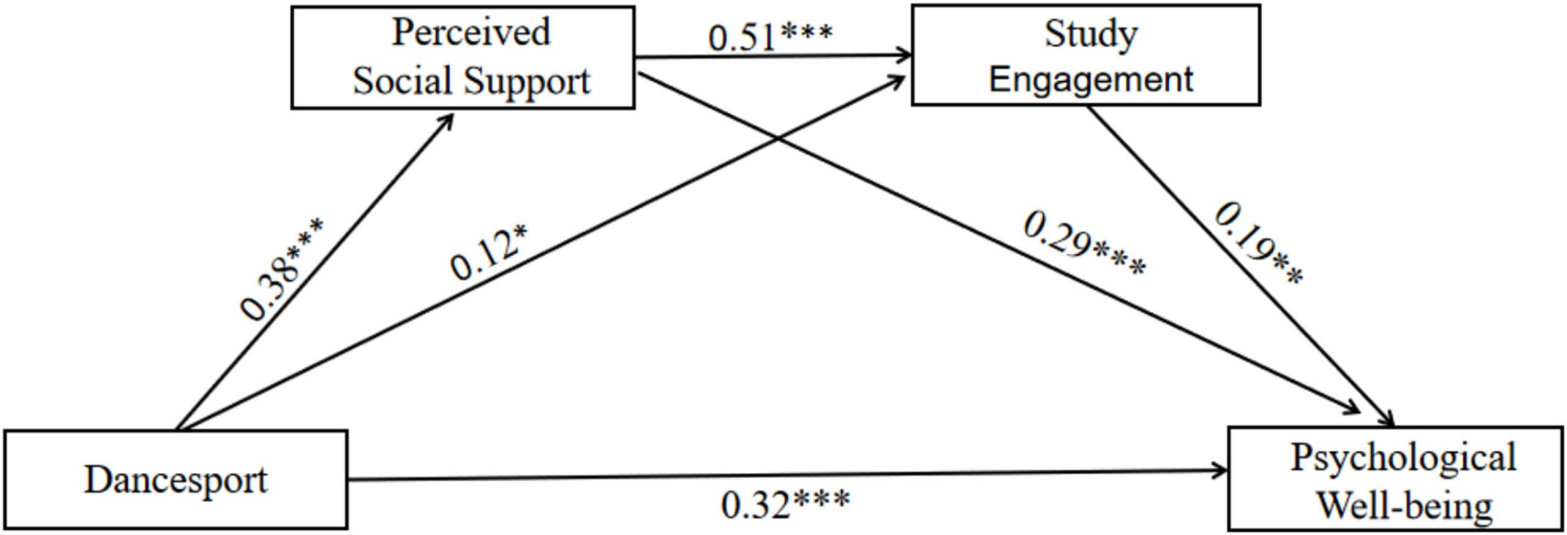
The effect of dancesport in psychological well-being of college students: chain mediation of perceived social support and study engagement.

Mediation effect tests showed that Bootstrap 95% confidence intervals for all paths did not contain 0, supporting the significance of the mediation effect (*p* < 0.05). The effect of dancesport on psychological well-being was 0.18 (95% CI = [0.09, 0.27]), accounting for 37.5% of the total effect. Therefore, H1 was validated; perceived social support mediated the effect of dancesport on psychological well-being, with a mediating effect of 0.11 (95% CI = [0.06, 0.17], excluding 0), accounting for 22.9% of the total effect. Therefore, H2 was validated; Dancesport is associated with psychological well-being through study engagement with a mediating effect of 0.08 (95% CI = [0.03, 0.13], excluding 0), accounting for 16.7% of the total effect. Therefore, H3 was validated; dancesport had a chain-mediated effect on perceived social support and study engagement through perception, with a mediation effect of 0.037 (95% CI = [0.015, 0.062], excluding 0), accounting for 7.7% of the total effect. Therefore, H4 was validated. The strongest individual mediation effect (22.9%) was found for perceived social support, indicating that social support was the core mechanism; the chain mediation pathway accounted for 7.7% of the total effect, but revealed the progressive role of “social resources → academic adaptation → happiness.” The specific results are shown in Table 4 and Figure 2.

**Table 4:** Chain mediation effect test of perceived social support and study engagement on dancesport and psychological well-being.

Type of effect	Value of effect	BootSE	95% CI		Percentage
			LLCI	ULCI	
Direct effect	0.18	0.06	0.09	0.27	37.5%
Perceived social support (alone)	0.11	0.03	0.06	0.17	22.9%
Study engagement (alone)	0.08	0.02	0.03	0.13	16.7%
Chain mediation	0.037	0.01	0.015	0.062	7.7%
Indirect total effect	0.30	0.05	0.21	0.40	62.5%
Total effect	0.48	0.05	0.39	0.57	100%

BootSE, Bootstrap Standard Error; LLCI/ULCI, Lower/Upper Limit of the 95% Confidence Interval. The confidence interval that does not contain zero indicates a significant effect.

## 4 Discussion

This study extends previous literature on physical activity and well-being by specifically investigating the underlying mechanisms in the context of dancesport, an activity that uniquely integrates physical exertion, artistic expression, and social interaction. While prior research has established the benefits of general exercise, our findings reveal a distinct chain mediation pathway—through perceived social support and subsequently study engagement—that may be particularly salient for dancesport. The synergistic nature of these components in dancesport likely fosters a richer accumulation of psychological and social resources compared to more solitary or non-artistic forms of exercise, offering a more nuanced explanation for its positive effects. In this study, we systematically explored the mechanism of dancesport on college students’ psychological well-being by constructing a chain mediation model, and revealed the dual mediation effects of “perceived social support” and “study engagement.” The results of the study not only verified the direct effect of dancesport on psychological well-being (H1), but also elucidated its indirect pathway through perceived social support and study engagement (H2, H3), and for the first time, proposed the chain intermediary effect of “dancesport → perceived social support → study engagement → psychological well-being” (H4). These findings have important theoretical implications for the fields of positive psychology, educational psychology, and physical fitness.

### 4.1 Relationship between physical dancesport and psychological well-being

The study found a significant positive correlation between dancesport participation and psychological well-being (r = 0.42, *p* < 0.01). This finding aligns with prior research (Cheng, 2021) and can be interpreted through the unique amalgamation of physical, artistic, and social elements in dancesport (Lu, 2023). This multidimensional nature may facilitate self-growth and environmental mastery, which are core dimensions of Ryff’s psychological well-being model. For instance, consistent with the notion of mastery experiences (Bandura, 1997), the process of acquiring dance skills likely enhances self-acceptance. Furthermore, the collaborative and expressive aspects of the activity can strengthen interpersonal relationships and foster a clearer sense of purpose in life (Lu, 2023). Research has shown that dancesport reduces anxiety levels by releasing endorphins, (Biddle and Asare, 2011). In addition, its artistic expressive properties contribute to self-efficacy (Bandura, 1997), which directly contributes to psychological well-being. This is consistent with Ryff’s multidimensional theory of psychological well-being, which suggests that individuals achieve higher levels of psychological fulfillment through physical activity for self-growth and environmental mastery (Ryff, 1989). Adolescents who regularly participate in physical activity have higher psychological well-being compared to adolescents who are often sedentary, and their psychological well-being decreases when regular exercise is transformed into regular sedentary activity (Sánchez-Oliva et al., 2020). Based on the above analysis further confirms that practicing dancesport is associated with enhanced self-confidence, improved aesthetics, and better physical and mental health among college students, which has a positive impact on college students’ psychological well-being and is conducive to enhancing college students’ psychological well-being. The positive association between dancesport and psychological well-being is particularly noteworthy within the context of the high academic pressure and competitive environment experienced by Chinese university students. In such a setting, dancesport may serve as a structured and enjoyable respite from academic demands, providing a crucial outlet for stress relief and self-expression that contributes uniquely to overall well-being.

### 4.2 Mediating role of perceived social support and study engagement

Perceived social support was found to play an important role between dancesport and psychological well-being. There was a positive correlation between perceived social support and psychological well-being (r = 0.47, *p* < 0.01). The mediating role of perceived social support validates the social support buffer hypothesis (Cohen and Wills, 1985). Social support, as the emotional support given by others, conveys positive beliefs and strength to the individual (Zhang et al., 2021). This aligns with the social support buffer hypothesis, as individuals with high perceived support are likely to appraise life challenges as less threatening and feel more confident in their ability to cope, thereby enhancing their well-being (Caserta et al., 2016). The group interaction characteristics of dancesport (e.g., partner cooperation, team practice) enhance college students’ perceptions of support from family, friends, and teachers (Zimet et al., 1988), which can alleviate stress, enhance emotion regulation (Thoits, 1983), and indirectly promote psychological well-being. This pathway has the highest percentage (22.9%), highlighting the centrality of social support in college students’ psychological well-being. Based on the above analysis, it can be concluded that perceived social support is associated with the relationship between dancesport and psychological well-being in a mediating role, and also has a facilitating effect on psychological well-being.

Study engagement was found to play a positive role between dancesport and psychological well-being. There was a positive correlation between study engagement and psychological well-being (r = 0.33, *p* < 0.01) The mediating role of study engagement reveals the transfer effect of physical activity on the academic domain (Fredrickson, 2001). Study engagement exhibits a sustained, positive emotional state filled with positive affect, which construct theory suggests can extend an individual’s sequence of momentary thinking activities to expand enduring personal resources such as stress coping and psychological well-being (Kerres Malecki and Kilpatrick Demary, 2001; Zhang, 2012). The focus and goal-oriented behaviors required for dancesport may translate into energy, dedication, and focus in learning (Schaufeli et al., 2002), and study engagement further enhances well-being through an enhanced sense of meaning (Seligman, 2011). This finding extends the applicability of the study engagement theory by suggesting that non-academic activities are equally capable of stimulating intrinsic motivation in students. Based on the above analysis, it can be concluded that study engagement is associated with the relationship between dancesport and psychological well-being in a mediating role, as well as contributes to psychological well-being.

### 4.3 Chain mediating role of perceived social support and study engagement

This study verified the positive chain-mediated effect of perceived social support and study engagement on psychological well-being in dancesport. Dancesport is associated with a social support environment that may help dancers establish positive social relationships and strengthen their psychological resilience. This study offers a new perspective on understanding and further exploring the relationship between dancesport and psychological well-being. Bandura’s social cognitive theory posits that behavior, human factors, and environmental factors are actually interrelated and interactive determinants (Bandura, 1978). Research has shown that support from family, peers, and teachers can promote students’ study engagement. In states of student anxiety, high support levels can help reduce anxiety and promote study engagement (Zhang, 2022). High levels of social support can alleviate students’ loneliness and enhance subjective well-being (Zhang et al., 2012), and influence study engagement through college students’ subjective support perception, objective support conversion, and support utilization (Zhang, 2015). After participating in social dancing, participants’ emotional levels increased (Wang, 2022). High levels of perceived social support make dancers feel supported and encouraged, enhancing their learning motivation and confidence. This positive social support further promotes dancers’ learning engagement, making them more active in dance learning and training. High learning engagement gives dancers more sense of achievement and satisfaction in dance learning, thereby improving their psychological well-being. Through enhancing perceived social support, Dancesport is associated with students’ study engagement, forming the “social resources → academic adjustment → happiness” progressive pathway. Although this pathway accounts for a small proportion (7.7%), it reveals the synergistic effect of social support and learning behavior and emphasizes the importance of the multi-level dynamic process of psychological health.

### 4.4 Theoretical contributions and practical implications

#### 4.4.1 Theoretical contributions

This study contributes to multiple theoretical domains by validating the chained mediating role of “perceived social support” and “study engagement” between ballroom dancing and psychological well-being. First, it expands the application of positive psychology in sports by revealing the complex mechanisms through which artistic and social activities enhance well-being—beyond merely physiological pathways. Second, it enriches educational psychology theory by confirming that social resources gained from non-academic activities (Dancesport) can positively transfer to academic domains (study engagement), forming a virtuous “social-academic-psychological” cycle. Finally, this study provides robust empirical support for the “sports-arts integration” health promotion model, emphasizing the potential superiority of multidimensional integrated interventions over isolated physical activities.

#### 4.4.2 Practical implications

The findings of this study offer valuable practical implications for supporting student development and campus wellness initiatives in higher education. University administrators and educators can.

Integrate Dancesport into public physical education courses or mental health promotion programs as an effective activity-based intervention to enhance students’ psychological well-being, social adaptation, and academic motivation.

Emphasize partner collaboration and team activities in course design to maximize social support benefits.

Encourage student clubs to organize Dancesport activities, creating a “psychological buffer” space rich in supportive interpersonal relationships beyond academic pressures.

Counselors and mental health educators can guide students exhibiting social withdrawal or academic burnout tendencies toward such activities, aiming to indirectly improve their learning performance and overall well-being by enhancing their perception of social support.

## 5 Research limitations and future prospects

(1) The data of this study only came from three universities in eastern China, and needs to be extended to different regions, cultural backgrounds and non-dance participants in the future to verify the generalizability of the findings. (2) The cross-sectional design of this study and the establishment of a chain mediation model to analyze the effects of perceived social support and study engagement on psychological well-being under the mediating effect of dances in sports, found the relationship between variables, which provides theoretical support for empirical research, but it is still necessary to explore the causal relationship between study variables through longitudinal tracking or experimental intervention studies (e.g., randomized controlled trials). Moreover, due to the cross-sectional design, bidirectional relationships cannot be ruled out. For example, students with higher psychological well-being might be more likely to participate in dancesport. Future longitudinal studies are needed to clarify the causal directions and confirm the mediation pathways.(3) This study relied solely on self-report measures, which are susceptible to biases such as social desirability (e.g., participants overreporting well-being or study engagement) and recall bias. Although we employed statistical and procedural controls (e.g., anonymity, counterbalancing items), these cannot fully eliminate the potential for inflated correlations between constructs. To overcome this limitation, future studies should adopt multi-method, multi-source assessment strategies. For instance, dancesport participation could be objectively quantified using accelerometers or heart rate monitors; behavioral coding of practice sessions could provide measures of engagement; and reports from peers or instructors could supplement self-reported perceptions of social support. (4) This study verified the chain mediation effect of perceived social support and study engagement, and did not examine other mediating variables: such as emotion regulation ability and self-efficacy, which may affect the chain path, and need to further explore the multiple mediating mechanisms. (5) The use of a convenience sample comprised solely of students enrolled in dancesport courses poses a limitation to the generalizability of our findings. This sampling approach likely introduces self-selection bias, as students who choose to take dancesport may systematically differ from their peers by having, for instance, initially higher levels of extraversion, social support, or psychological well-being. Consequently, the observed associations might overestimate the effects for the general student population. To robustly establish the unique contribution of dancesport, future research should employ designs that include comparison groups, such as students participating in other forms of exercise and non-participating controls. This would allow for a more direct test of whether the benefits are specific to the multidimensional (artistic + social) nature of dancesport.

Future research could include more variables to analyze the moderating role of individual characteristics (e.g., personality traits, exercise experience) on the mediating effects; and combine mixed research methods (quantitative + qualitative) to gain a deeper understanding of the process of students’ experiences and psychological changes in their participation in dancesport.

## 6 Conclusion

This study elucidates the mechanisms by which dancesport enhances psychological well-being in Chinese college students, confirming a direct effect (H1) and significant mediation through perceived social support (H2) and study engagement (H3). Crucially, it establishes a sequential pathway: dancesport → perceived social support → study engagement → psychological well-being (H4). This reveals dancesport not merely as physical exercise, but as a multifaceted intervention that builds social resources to foster academic engagement and, ultimately, well-being.

Consequently, integrating dancesport into university wellness programs offers a potent strategy to simultaneously promote physical, social, and academic vitality. By uncovering these specific psychosocial mechanisms, this study provides a robust empirical foundation for employing multidimensional activities like dancesport in holistic student development and mental health promotion strategies.

## Data Availability

The raw data supporting the conclusions of this article will be made available by the authors, without undue reservation.
